# Supplementary material for: Social Media Usage among Dental Undergraduate Students—A Comparative Study
Source: Healthcare (Basel). 2021 Oct 20;9(11):1408. doi: 10.3390/healthcare9111408 (PMC8625251; doi:10.3390/healthcare9111408)
Supplement: Supplementary file 1 [file healthcare-09-01408-s001.zip › healthcare-1385433-supplementary.pdf]

Questionnaire on Social Media usage by Dental Students

1. What dental school are you at?
   - o University of Helsinki
   - o University of Oulu
   - o Manipal University College Malaysia
   - o Faculty of Dentistry, University Malaya

2. What is your sex?
   - o Male
   - o Female

3. What is your age?
   - o 20 or younger
   - o 21-23
   - o 24-26
   - o 27-29
   - o 30-35
   - o 35 and above

4. How do you identify yourself? (In the Finnish version, this wase replaced by the following text (in Finnish): The question is related to ethnic background and does not concern Finnish students)
   - o Malay
   - o Chinese
   - o Indian
   - o Other Bumiputera
   - o Other (please specify)

     ………………………..

5. What year of dental school are you currently in?
   - o First
   - o Second
   - o Third
   - o Fourth
   - o Fifth
   - o Other (please specify) …………………

6. How familiar are you with each of the following social media services?

	Never heard of it	Heard of it; not sure of its purpose	Familiar with its purpose	Very familiar with its purpose
○ Facebook				
○ Twitter				
○ LinkedIn				
○ YouTube				
○ Google+				
○ Pinterest				
○ Tumblr				
○ Instagram				
○ WhatsApp				
○ Snapchat				
○ Jodel				
○ TikTok				
○ Telegram				
○ WeChat				
○ Weibo				

If there are other social media services with which you are familiar, please list and rate your familiarity here.

……………………………………………………………………………………………………….…………..…

7. How competent are you at using each of the following social media services?

	Not at all	Beginner	Competent	Highly competent
○ Facebook				
○ Twitter				
○ LinkedIn				
○ YouTube				
○ Google+				
○ Pinterest				
○ Tumblr				
○ Instagram				
○ WhatsApp				
○ Snapchat				
○ Jodel				
○ TikTok				
○ Telegram				
○ WeChat				
○ Weibo				

If there are other social media services that you use, please list and rate your competence here:

……………………………………………………………………………..……………………

8. How often do you use each of the following social media services?

	Never	Occasionally (not usually more than once a month)	Regularly (not daily but at least weekly)	Frequently (daily)	Very frequently (several times a day
○ Facebook					
○ Twitter					
○ LinkedIn					
○ YouTube					
○ Google+					
○ Pinterest					
○ Tumblr					
○ Instagram					
○ WhatsApp					
○ Snapchat					
○ Jodel					
○ TikTok					
○ Telegram					
○ WeChat					
○ Weibo					

If there are other social media services that you use, please list and rate how often you use them here:

……………………………………………………………………………………………...…………..

9. How important is each of the following factors in encouraging you to use social media?

	Not at all important	Somewhat important	Very important	I don't use social media
To stay in touch with current friends and family members				
To connect with old friends, I have lost touch with				
To connect around a shared hobby				
To communicate about issues relating to dental training				

10. How much do the following reasons prevent you from using social media?

	Not at all	Somewhat	Very much
Lack of knowledge			
Lack of time			
Lack of interest			
Lack of perceived value			
Concern about harm to professional image			

Other (please specify): ………………………………………………………………………….………..

11. Approximately how many hours **A WEEK** do you spend using social media?

- o Do not use)
- o Less than 5 hours
- o 6-10 hours
- o 11-15 hours
- o 16-20 hours
- o More than 20 hours

12. Approximately how many hours **A WEEK** do you spend using the following social media services as **part of your dental education**?

	Do not use	Less than 1 hour	1-5 hours	6-10 hours	11-15 hours	16-20 hours	More than 20 hours
o Facebook							
o Twitter							
o Linked In							
o YouTube							
o Google+							
o Pinterest							
o Tumblr							
o Instagram							
o Whatsapp							
o Snap chat							
o Jodel							
o Tik Tok							
o Telegram							
o WeChat							
o Weibo							
